# Cystatin C Falsely Underestimated GFR in a Critically Ill Patient with a New Diagnosis of AIDS

**DOI:** 10.1155/2016/9349280

**Published:** 2016-05-17

**Authors:** Caitlin S. Brown, Kianoush B. Kashani, Jeremy M. Clain, Erin N. Frazee

**Affiliations:** ^1^Hospital Pharmacy Services, Mayo Clinic, 200 First Street SW, Rochester, MN 55905, USA; ^2^Division of Pulmonary and Critical Care Medicine, Mayo Clinic, 200 First Street SW, Rochester, MN 55905, USA; ^3^Division of Nephrology and Hypertension, Mayo Clinic, 200 First Street SW, Rochester, MN 55905, USA

## Abstract

Cystatin C has been suggested to be a more accurate glomerular filtration rate (GFR) surrogate than creatinine in patients with acquired immunodeficiency syndrome (AIDS) because it is unaffected by skeletal muscle mass and dietary influences. However, little is known about the utility of this marker for monitoring medications in the critically ill. We describe the case of a 64-year-old female with opportunistic infections associated with a new diagnosis of AIDS. During her course, she experienced neurologic, cardiac, and respiratory failure; yet her renal function remained preserved as indicated by an eGFR ≥ 120 mL/min and a urine output > 1 mL/kg/hr without diuresis. The patient was treated with nephrotoxic agents; therefore cystatin C was assessed to determine if cachexia was resulting in a falsely low serum creatinine. Cystatin C measured 1.50 mg/L which corresponded to an eGFR of 36 mL/min. Given the >60 mL/min discrepancy, serial 8-hour urine samples were collected and a GFR > 120 mL/min was confirmed. It is unclear why cystatin C was falsely elevated, but we hypothesize that it relates to the proinflammatory state with AIDS, opportunistic infections, and corticosteroids. More research is needed before routine use of cystatin C in this setting can be recommended.

## 1. Introduction

In critically ill patients, serum creatinine suboptimally predicts glomerular filtration rate (GFR) because of its delayed rise during acute injury and the myriad of confounders which influence its concentration including skeletal muscle mass, dietary intake, interactions with vasopressors, antibiotics, and bilirubin and fluid shifts [1–4]. For this reason cystatin C, an alternate endogenous marker of GFR, may be more suitable. Cystatin C, a 13 kDa protein produced at a constant rate by all nucleated cells, is freely filtered by the glomeruli and, unlike creatinine, does not undergo tubular secretion [1, 4–6]. In stable chronic kidney disease (CKD) patients, cystatin C in combination with creatinine better predicted measured GFR than either biomarker alone [2]. Unfortunately, studies on the performance of this GFR surrogate in the critically ill are limited and conflicting [7, 8]. In the intensive care unit (ICU), several complicating factors such as acute inflammatory states and conditions or treatments that impact cell turnover may precipitate elevations in cystatin C independent of changes in underlying GFR, thereby rendering this biomarker of questionable value [5].

Critically ill patients diagnosed with human immunodeficiency virus (HIV) represent a group of individuals in whom interpretation of GFR is not only essential but also difficult. Indeed, patients diagnosed with HIV exhibit a 10-fold greater risk of developing end-stage renal disease (ESRD) than individuals without HIV [9]. HIV-positive patients are often cachectic, thereby reducing the sensitivity of serum creatinine for the detection of renal dysfunction. They also exist in a state of acute or chronic inflammation, therein complicating interpretation of cystatin C. Cystatin C, being an inflammatory marker, has been shown to decrease after initiation of antiretroviral therapy [10, 11]. Only limited evidence exists to indicate which of these GFR surrogates should be used in critically ill HIV-positive patients [9, 12, 13]. We report our experience in a treatment-naïve patient with a new diagnosis of acquired immunodeficiency syndrome (AIDS) in whom cystatin C falsely underestimated GFR.

## 2. Case Report

A 64-year-old Caucasian female (height 152 cm, admission weight 55.2 kg) was transferred to a tertiary care center ICU from an outside hospital for worsening acute hypoxemic respiratory failure. The patient's known past medical history included Crohn's disease most recently treated with mesalamine and prednisone 60 mg daily. Work-up at the outside hospital revealed* Pneumocystis jiroveci* pneumonia (PJP) and the patient was preliminarily treated with atovaquone and a single dose of intravenous pentamidine due to a reported sulfa allergy. Upon admission to the ICU, her initial serum creatinine was 0.4 mg/dL (Table 1). At our institution, serum creatinine is measured using a standardized enzymatic assay on a Roche Cobas chemistry analyzer (c701 or c501) (Roche Diagnostics, Indianapolis, USA), and in patients receiving intravenous catecholamines, known to interfere with enzymatic assays, an IDMS traceable Roche rate Jaffe creatinine assay is used. Cystatin C was measured using an immunoturbidimetric assay (Gentian, Moss, Norway) that was traceable to an international reference material [14–16].

By ICU day 4, in addition to PJP, she was diagnosed with herpes simplex virus pneumonitis (HSV), cytomegalovirus (CMV) viremia with pulmonary involvement, and acalculous cholecystitis. Her antimicrobial regimen included cefepime, metronidazole, and vancomycin to target pulmonary and intra-abdominal sources. She was also desensitized to sulfamethoxazole-trimethoprim and treated with 15 mg/kg/day in divided doses in conjunction with steroids for PJP, initiated on ganciclovir 5 mg/kg twice daily for treatment of HSV and CMV, and started on cytomegalovirus immune globulin 150 mg/kg every other day due to a CMV viral load > 9,100,000 IU/mL. Over the course of the subsequent days, the patient experienced progressive circulatory and respiratory failure; yet her renal function remained preserved as indicated by a stable serum creatinine of <0.2–0.4 mg/dL (creatinine clearance ≥ 120 mL/min) and urine output consistently in excess of 1 mL/kg/hr without diuretic support.

On the eighth day of ICU admission, the patient was found to be HIV-positive, with a CD4 count of 0 cells/mcL, and further work-up revealed central nervous system (CNS) and colon involvement of the CMV. Foscarnet, a nephrotoxic antiviral suggested by the guidelines for dual coverage of central nervous system CMV involvement, was to be started in conjunction with ganciclovir [17, 18]. Given the patient's cachexia, creatinine was suspected to be an unreliable marker of GFR, therefore prompting cystatin C to be checked in order to assist with dosing and monitoring of foscarnet and other antimicrobials. Cystatin C measured 1.50 mg/L, which corresponded to an estimated GFR of 36 mL/min, considerably lower than the GFR predicted by creatinine. Due to the degree of the disparity, other surrogates of GFR were explored. A sulfamethoxazole peak level on full-dose therapy was found to be 136 mcg/mL, which is within the target range of 100–150 mcg/mL and suggestive of a GFR approximated by the serum creatinine-based estimate [19]. An 8-hour urine creatinine clearance was also collected and indicated a GFR > 120 mL/min (Table 2) [20]. As cystatin C was the only marker indicating renal insufficiency, this was disregarded and medications were dosed according to a GFR > 120 mL/min. Serial monitoring of the creatinine, cystatin C, sulfamethoxazole concentration, and 8-hour urine creatinine clearance was performed throughout the ICU course and consistently suggested that the serum creatinine better estimated the patient's true GFR than the serum cystatin C. Unfortunately, despite aggressive supportive measures and initiation of antiretroviral therapy, the patient failed to meaningfully improve. She was transitioned to comfort measures and died on hospital day 16.

## 3. Discussion

Serum creatinine has historically been the standard to estimate GFR and optimize medication dosing in HIV-positive patients, but in recent years cystatin C has been shown to be an equivalent or more reliable alternative [1, 9, 12]. Our case illustrated a >60 mL/min difference in estimated GFR when calculated with serum creatinine versus serum cystatin C. An accurate assessment of GFR was imperative in this patient as she exhibited a high severity of illness requiring administration of multiple nephrotoxic medications with narrow therapeutic indices. The patient had numerous factors that could have contributed to the inaccuracy of serum creatinine, including cachexia and its unreliability in the acute setting. However, given that the sulfamethoxazole concentrations were found to be in the therapeutic range and that the creatinine clearance was found to exceed 120 mL/min on the 8-hour urine collections, the patient's true GFR appears to have been better estimated by the serum creatinine than by the serum cystatin C either alone or in combination via the CKD-EPI_creatinine-cystatin  C_ equation.

Patients with HIV have been thought to be potentially suitable candidates for the use of cystatin C due to their unpredictable muscle mass [9, 12]. In a study by Seape et al., cystatin C based equations to measure GFR were more accurate than creatinine-based questions in an HIV-treatment-naïve South African population [1]. Another study, by Driver et al., looked at a cohort of HIV-infected women and found that GFR estimated by cystatin C was more sensitive in detecting reduced renal function than creatinine. However, there is limited evidence to support the use of cystatin C in critically ill HIV-positive patients. The patient's cystatin C levels did decrease after initiation of antiretroviral therapy, as evident in Table 1.

It is unknown what factors could have obscured the relationship between the cystatin C measurement and underlying GFR in this patient. A previous study of non-GFR factors associated with cystatin C concentrations found diabetes mellitus, increased C-reactive protein, increased white blood cell count, and decreased albumin concentrations to be independently predictive of increased cystatin C [5]. Elevated cystatin C levels have also been associated with increased body mass index and inflammation [5, 21]. Certainly, cystatin C use has its limitations because it is produced by all nucleated cells; therefore, patients with heightened cell turnover may experience elevations in cystatin C due to underlying disease as opposed to true reductions in GFR [6, 21]. In a study of HIV-positive patients, nonuse of antiretrovirals, lower CD4 cell count, higher HIV RNA copies, and greater T-cell activation were associated with poor accuracy of cystatin C GFR estimates. These inaccuracies were not observed when GFR was calculated with creatinine [22]. We suspect that, in the present case, the elevated cystatin C reflected the widespread inflammation and T-cell activation associated with multiple opportunistic infections in the setting of a new diagnosis of AIDS, along with the effects of high-dose corticosteroid therapy. Although the CKD-EPI_creatinine-cystatin  C_ equation has been shown to be a better indicator of GFR in both HIV-positive and non-HIV patients than the CKD-EPI_creatinine_ or CKD-EPI_cystatin  C_ equations, few of the patients in these studies were critically ill and in the midst of disseminated infections [2, 13].

In this case of a patient with a new diagnosis of AIDS, cystatin C falsely underestimated the patient's renal function. Further studies are needed to determine the role of cystatin C in medication use and monitoring in critically ill patients, especially among those with HIV.

## Figures and Tables

**Table 1:** Measured serum GFR surrogates including creatinine, cystatin C, and sulfamethoxazole concentrations.

Hospital day^a^	Serum creatinine (mg/dL)	Cockcroft-Gault creatinine clearance (mL/min)	Cystatin C (mg/L)	CKD-EPI_cystatin C_ (mL/min)	Sulfamethoxazole^b^ peak level (mcg/mL)
1	0.4	>120	—	—	—
7	<0.2	>120	—	—	136
8	<0.2	>120	1.50	36	—
9	<0.2	>120	1.42	39	—
14	0.2	>120	1.11	54	84

^a^Hospital day 1 reflects the day of transfer from the outside facility to our center.

^b^Target peak range for sulfamethoxazole in PJP is 100–150 mcg/mL. Both of the measured levels were documented while the dose of sulfamethoxazole/trimethoprim was 15 mg/kg/day of the trimethoprim component administered thrice daily as appropriate for therapeutic dosing in individuals with an estimated creatinine clearance of ≥30 mL/min.

**Table 2:** Creatinine clearance following the 8-hour urine collections.

Hospital day^a^	Urine creatinine (mg/dL)	Serum creatinine (mg/dL)	Urine volume (mL)	Collection duration (hours)	Urine creatinine clearance^b^ (mL/min)
10	24	0.2	714	8	179
14	10	0.2	2132	8	222

^a^Hospital day 1 reflects the day of transfer from the outside facility to our center.

^b^Creatinine clearance (mL/min) = [urine creatinine × volume (in mL)]/[serum creatinine × (time (in hours) *∗* 60)].
